# High-efficiency chiral meta-lens

**DOI:** 10.1038/s41598-018-25675-3

**Published:** 2018-05-08

**Authors:** Benedikt Groever, Noah A. Rubin, J. P. Balthasar Mueller, Robert C. Devlin, Federico Capasso

**Affiliations:** John A. Paulson School of Engineering and Applied Sciences, Harvard University, Cambridge, MA USA

## Abstract

We present here a compact metasurface lens element that enables simultaneous and spatially separated imaging of light of opposite circular polarization states. The design overcomes a limitation of previous chiral lenses reliant on the traditional geometric phase approach by allowing for independent focusing of both circular polarizations without a 50% efficiency trade-off. We demonstrate circular polarization-dependent imaging at visible wavelengths with polarization contrast greater than 20dB and efficiencies as high as 70%.

## Introduction

Polarization, a fundamental characteristic of electromagnetic radiation, is manifest in almost all optical phenomena, from reflection and transmission at an interface, scattering by small particles, and the physics of atomic transitions^1^. Polarization-dependent effects are also manifest in many areas of optical technology, notably in polarization-resolved imaging^2^. Here it has attracted significant interest, as polarization-sensitivity provides contrast enhancement. This has found application in remote-sensing^2–4^, atmospheric science^4–6^, medicine^5–9^, and biological imaging^10–13^. Circular polarization states are of particular interest – acquisition of the circular component of light’s polarization may not be accomplished with simple linear polarizers alone, and thus represents the most challenging aspect of polarization measurement. Moreover, circular polarizations are of particular interest because of their interaction with the chiral structures abundant in biochemistry, and hold a certain fundamental significance in physics.

One substantial drawback of polarization imaging, and polarization optics generally, is that it typically requires relatively complex optical trains, consisting of multiple beam splitters, linear polarizers, and birefringent crystal waveplates (in order to obtain information about the circular polarization content of the light)^2^. In this realm, metasurfaces represent an important new opportunity, enabling the combination of multiple optical elements into a single component. The dimensions of the individual phase-shifting elements composing a metasurface may be adjusted, allowing for exquisite control over the phase imparted on orthogonal linear polarizations. This aspect enables the design of metasurfaces which may act in a polarization-dependent fashion. Moreover, metasurfaces are also extremely compact, consisting of flat, sub-wavelength arrays of microscopic phase elements^14–17^, and may be fabricated in a variety of material platforms depending on the wavelength regime of interest.

In recent years, metasurfaces and diffractive optics utilizing the so-called geometric phase have emerged as novel optical elements for addressing circular polarization states^18–20^. In the most common geometric phase-based scheme, a birefringent element (i.e., a half-wave plate) rotated at some angle *θ* causes left- and right-hand circularly polarized light to acquire equal and opposite phase shifts *ϕ*_LCP_ = +2*θ* and *ϕ*_RCP_ = −2*θ*, respectively, upon interaction with the phase-shifter^21–23^. In this way, any desired phase profile may be imparted on one circular polarization state; however, since the phase shifts are always equal and opposite, only one of the circular polarization states may be independently controlled with such a scheme.

This has some interesting and notable consequences. For example, if *ϕ*_LCP_(*x*,*y*) is a converging lens phase profile, *ϕ*_RCP_(*x*,*y*) is constrained to act as a diverging lens. This results in a 50% efficiency loss in any geometric phase device aimed at independently imaging both circular polarizations, even if two appropriate geometric phase designs are interlaced^10,24^. Moreover, the unfocused light forms an undesired background, lowering the signal-to-noise ratio of any circular polarization-discriminating measurement or image. Despite the advantage of geometric phase optical elements (such as ease of fabrication and simplicity), these effects threaten to limit their ultimate usefulness.

**Figure 1:**
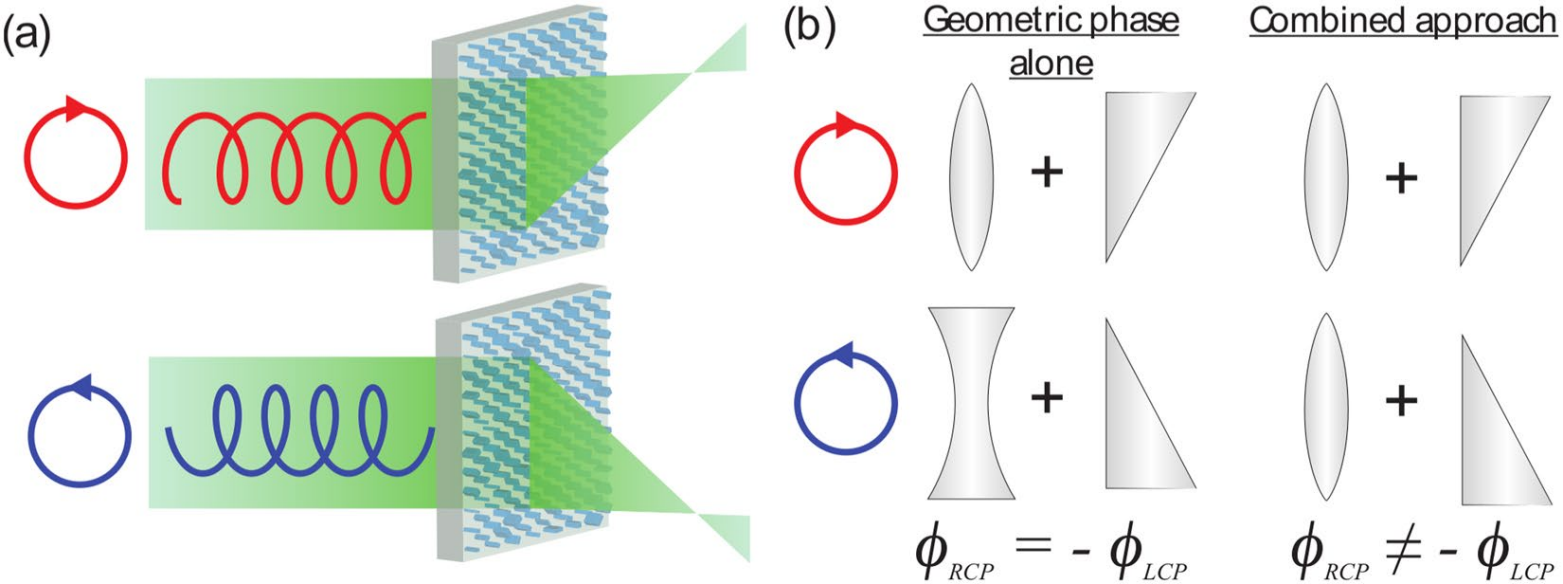
Chiral meta-lens principle. (**a**) Illuminated with LCP or RCP light, the meta-lens focuses an RCP - LCP image on two different positions. (**b**) Analogy demonstrating fundamental design differences with respect to previous chiral-lenses. A geometric phase lens designed for RCP has an equal and opposite phase profile for LCP. It focuses RCP to the right and defocus LCP to the left, here in analogy represented with a refractive lens and a wedge. The combined approach (propagation & geometric phase) allows focusing of LCP and RCP independently from each other.

In the present work, we demonstrate a metasurface lens focusing each circular polarization at different angles without the above-mentioned tradeoff (Fig. 1a). Figure 1b illustrates the merit of this approach. An off-axis lens is analogous to a superposition of the phase profiles of a lens and a wedge. If this phase profile is implemented using the geometric phase (left), one circular polarization will be focused off-axis, but when its handedness is reversed, the lens phase profile will act like a diverging lens and the slope of the wedge phase profile will change sign. Using the approach presented here, an off-axis lens may be designed truly focusing light to two off-axis positions, that is, by flipping the wedge without changing the lens from converging to diverging (right).

Our solution relies on a recently established general approach to metasurface polarization optics that does not rely on the geometric phase alone: by using a combination of geometric and propagation phases in tandem it is possible to impart fully arbitrary and independent phase profiles on the two orthogonal polarizations states^25,26^, including the two circular polarization states. This overcomes the symmetry constraint of geometric phase lenses, and a chiral lens that focusses the two polarizations in separate locations may be readily designed (Fig. 1a).

## Design

The Jones matrix *J* of a linearly birefringent waveplate imparting phases *ϕ*^+^ and *ϕ*^−^ independently on any two orthogonal input polarization states with Jones vectors ${\overrightarrow{\lambda }}^{+}=({\lambda }_{1}^{+},{\lambda }_{2}^{+})$ and ${\overrightarrow{\lambda }}^{-}=({\lambda }_{1}^{-},{\lambda }_{2}^{-})$ is given by^25^:

1 $$J=(\begin{array}{cc}{e}^{i{\varphi }^{+}}{({\lambda }_{1}^{+})}^{\ast } & {e}^{i{\varphi }^{-}}{({\lambda }_{1}^{-})}^{\ast }\\ {e}^{i{\varphi }^{+}}{({\lambda }_{2}^{+})}^{\ast } & {e}^{i{\varphi }^{-}}{({\lambda }_{2}^{-})}^{\ast }\end{array}){(\begin{array}{cc}{\lambda }_{1}^{+} & {\lambda }_{1}^{-}\\ {\lambda }_{2}^{+} & {\lambda }_{2}^{-}\end{array})}^{-1}$$

In the case of metasurfaces, a birefringent phase shifting element implementing J must be found. J’s eigenvectors specify the angular orientation of the required element. The phases of its eigenvectors specify the phase shifts required for linear polarized light along its symmetry axes. The phase shift for linear polarization along each symmetry axis can be controlled through the geometric dimensions of each element. Geometries best matching these required phase shifts may be drawn from a library of known elements with roughly equal, high transmissivity. The above method can be understood as a unification of the propagation and geometric phases in a single element such that two independent lens profiles can be imparted. Previous designs relied on multiplexing^10,24^.

For the present chiral meta-lens, the two orthogonal polarization states are given by ${\overrightarrow{\lambda }}_{{\rm{RCP}}}=\frac{1}{\sqrt{2}}\mathrm{(1,}\,i)$ and ${\overrightarrow{\lambda }}_{{\rm{LCP}}}=\frac{1}{\sqrt{2}}\mathrm{(1,}\,-i)$. We desire to focus opposite circular polarizations to two points with equal focal lengths but equal and opposite angular displacements from the optic axis. In general, the location of the RCP and LCP foci could be fully arbitrary. The phase profiles for each polarization (Supp. Note 1) are then given by:

2 $${\varphi }_{{\rm{LCP}}}=-\frac{2\pi }{{\lambda }_{0}}[\sqrt{{x}^{2}+{y}^{2}+{f}^{2}-2xf\,\sin \,\theta }-f]$$

3 $${\varphi }_{{\rm{RCP}}}=-\frac{2\pi }{{\lambda }_{0}}[\sqrt{{x}^{2}+{y}^{2}+{f}^{2}+2xf\,\sin \,\theta }-f]$$

Here, *f* denotes the desired focal length, *θ* denotes the off-axis angle of the lens, *λ*_0_ is the design (free-space) wavelength, and *x* and *y* are Cartesian spatial coordinates of the lens. These phase profiles can be understood as a hyperbolic lens phase profile merged with an equal and opposite gradient term for RCP and LCP (Fig. 1b, and Supp. Note 2).

In this work, we implement the above design using rectangular-shaped pillar elements, fabricated in TiO2. Neither choice is fundamental: in principle, any phase shifter geometry with two perpendicular mirror symmetry axes could suffice. TiO2 was chosen to target the ubiquitous visible range where TiO2 has low losses and high index, though given a proper material platform these concepts apply at any frequency.

**Figure 2:**
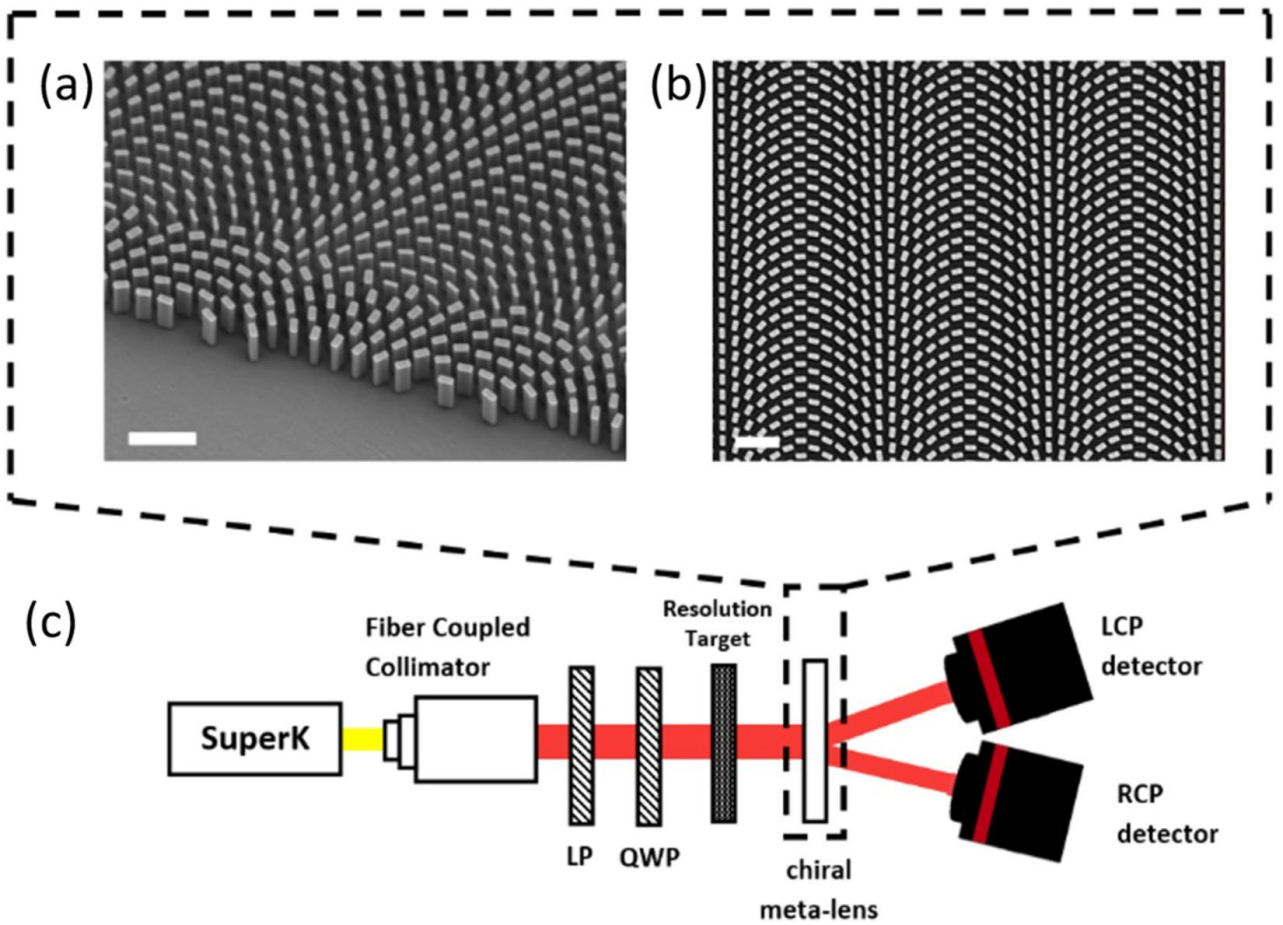
Device and measurement setup. (**a**) Top-side view of the SEM micrograph picture at the edge of the metasurface lens. Scale bar: 1 (**b**) Top-view SEM micrograph picture close to the center of the chiral meta-lens. Scale bar: 1 (**c**) The 1951 USAF resolution test chart illuminated with a supercontinuum laser. The polarization is controlled with a linear polarizer LP and a quarter-wave plate QWP. The sample focuses the LCP and RCP image of the resolution test chart on detector.

In choosing element parameters, we draw from an FDTD-simulated library of pillars with dimensions in *x* and *y* ranging between 50 and 250 nm, with a height fixed at 600 nm. The variation in the dimension from 50 to 250 nm provided the full phase coverage from 0 to 2*π* for both linear polarizations simultaneously. The pillars, fabricated on a 500-thick SiO2 substrate with a previously-reported fabrication process^27^, had a 350 nm nearest-neighbor separation on a hexagonal lattice. The lens is designed for *λ*_0_ = 532 nm, with *f* = 18 mm and *θ* = 8°. The diameter of the meta-lens as-fabricated is 1.8 mm, yielding a numerical aperture (NA) of 0.05. At such low NA, the imaging system does not need to be designed for a specific image and object distance but can still focus diffraction-limited^28^. Electron micrographs of the fabricated chiral meta-lens are shown in Fig. 2a,b.

## Characterization

In a first measurement, we sought to characterize the chiral imaging capabilities of the chiral meta-lens. A 1951 USAF resolution test chart is illuminated by a fiber-coupled super-continuum laser source whose wavelength can be varied throughout the visible range. Additionally, as depicted in Fig. 2c, the light passes through a linear polarizer (LP) and a broadband quarter-wave plate (QWP) before reaching the resolution target, allowing the incident polarization to be varied.

**Figure 3:**
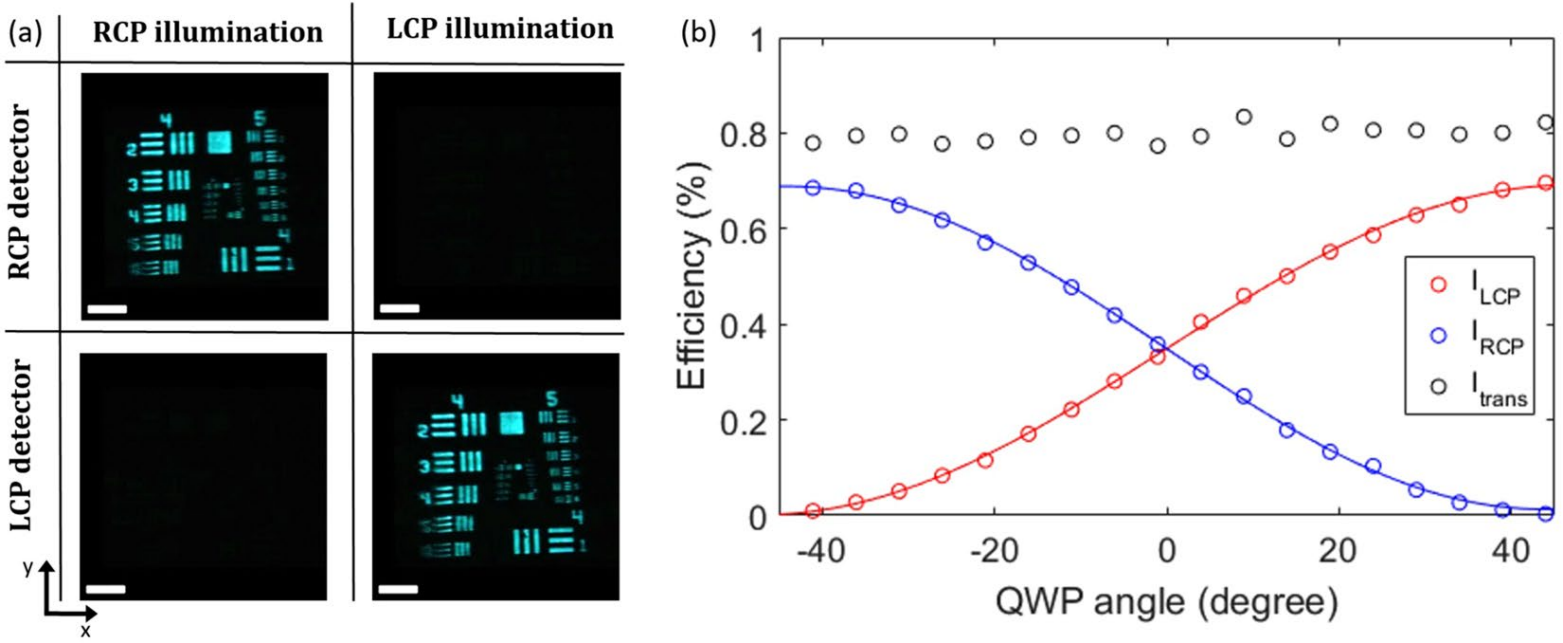
Device Performance. (**a**) RCP and LCP images taken of the 1951 USAF resolution test chart with the chiral meta-lens for RCP and LCP illumination at 500 nm. Scale bar: 180. In group 4 the bars have a spacing and width ranging from 31.25 to 17.54, in group 5 from 15.63 to 8.77. (**b**) Focusing efficiency for RCP (*I*_RCP_), LCP (*I*_LCP_) and transmission efficiency (*I*_trans_) at different quarter wave plate (QWP) angles for 500 nm illumination. The transmission efficiency through the chiral meta-lens is about 80% from which LCP and RCP have a combined focusing efficiency of 70%. Most of the remaining light couples to the 0th order while higher orders can be neglected. The data for LCP and RCP focusing efficiency is fitted with: ${I}_{{\rm{RCP}}}=0.3377\,\sin \,(\,-\,0.0359\theta -\mathrm{0.0065)}+0.3509$ and ${I}_{{\rm{LCP}}}=0.3451\,\sin (\,-\,0.0334\theta +\mathrm{3.1328)}+0.3463$, here *θ* is the QWP angle in degrees.

The images produced by the metasurface lens under different circular polarizations are shown in Fig. 3a. For linearly polarized light an equally bright image would appear on both detectors since it contains an equal proportion of LCP and RCP. The smallest bars in group 5 have a spacing and width of 8.77 which is larger than the diffraction limit of the chiral meta-lens: $d={\lambda }_{0}\mathrm{/2}NA$ = 5 μm. Due to the smaller size of the bars on the resolution test chart, the intensity contrast is lower. The spatial resolution along the x-axis is more sensitive to chromatic aberrations than the spatial resolution along the y-axis due to the grating term ±2*x f* sin*θ* in the phase profile. This effect can be seen in the different sharpness between the horizontal and vertical bars in the image, because of the bandwidth of the super-continuum laser (10 nm).

In a second measurement, we characterized the LCP - RCP focusing efficiency for different polarizations. For this measurement the resolution test target in setup Fig. 2b was removed, and optical power meters were placed at the LCP and RCP foci. The incident polarization is changed by rotating the QWP. The LCP - RCP focusing efficiency, *I*_LCP_ − *I*_RCP_, which is defined as the ratio of the intensity in the respective focal spot over the incident intensity across the aperture of the meta-lens, was measured for various QWP angles. The QWP angle, *θ*, is defined as the angle between the incident linear polarization set by the polarizer and the QW plate fast axis, *θ* = 0°. Light incident on the chiral meta-lens is linearly polarized with vertical orientation and then focused as two beams of opposite CP and equal intensity. As |*θ*| increases from 0° to 45° the incident beam is elliptically polarized with varying ratios of LCP and RCP amplitudes, leading to different measured intensities by the two detectors, shown in Fig. 3b.

The RCP and LCP focusing efficiency have the expected sinusoidal form with a maximum of 70%. This is significantly higher than the fundamental 50% efficiency trade-off imposed by geometric phase designs. Meta-lenses based on the geometric phase, in contrast, have been reported at a maximum focusing efficiency of 24%, owing to fundamental limits and practical constraints^10^. The transmission efficiency, *I*_trans_, defined as transmitted intensity through the meta-lens over the incident intensity across the aperture of the meta-lens, was measured by bringing the power meter close to the meta-lens, so that all the diffraction orders were captured. The transmission efficiency is approximately 80% and polarization invariant. The coupling into the 0th order is also polarization insensitive and stays around 10% (Supp. Note 3). It originates from the discretization of the phase profile^29^ and fabrication imperfections that affect transmission and phase.

**Figure 4:**
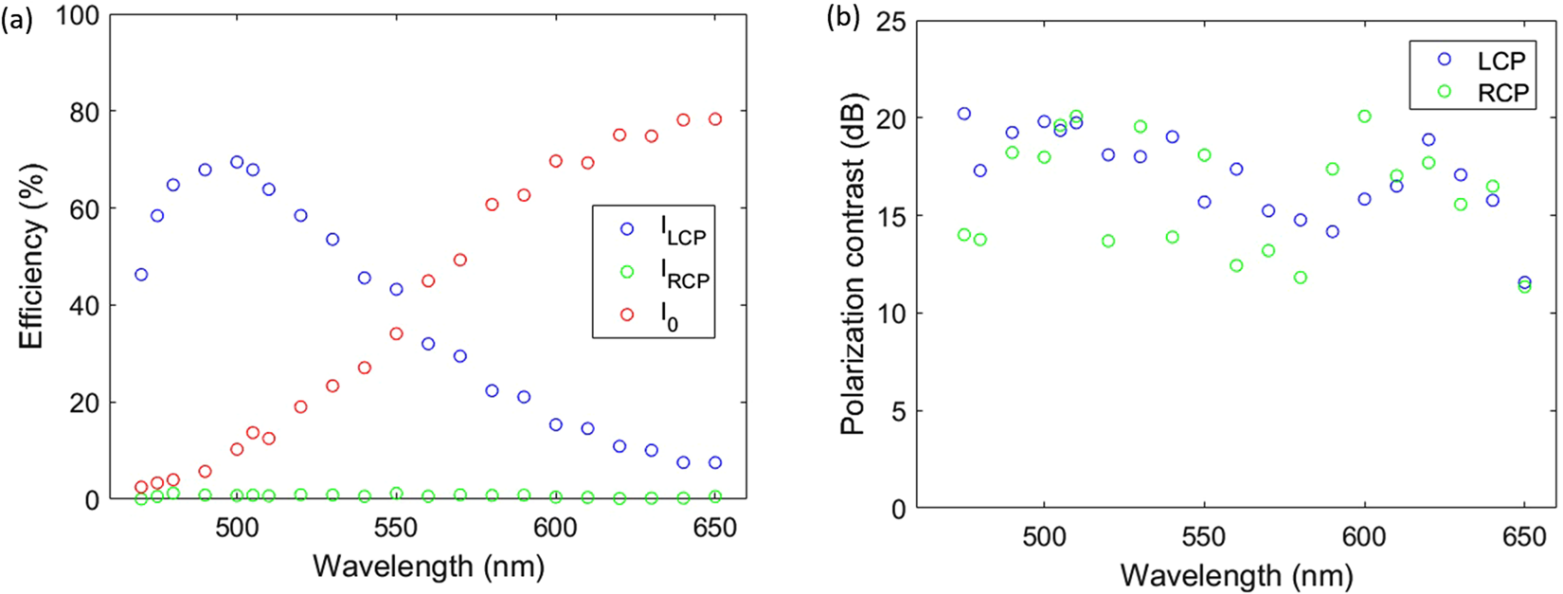
Broadband properties. (**a**) Focusing efficiency for LCP - RCP and 0th order coupling loss for LCP illumination at different wavelengths. The LCP focusing efficiency is peaked at 500 nm. The intensity in the 0th order increases gradually from to 0% to almost 80% in the wavelength range 470 nm to 650 nm. (**b**) The polarization contrast for RCP - LCP from 470 nm to 650 nm.

Though the lens is designed for a single wavelength, its imaging capabilities are relatively broadband (Supp. Note 4). We observe that the LCP focusing efficiency (shown in Fig. 4a) and the RCP focusing efficiency (Supp. Note 5) peak at 500 nm. We believe this offset from the design wavelength (532 nm) can be explained through fabrication imperfections in width, length and height of the nanopillars.

The polarization contrast, shown in Fig. 4b, is defined as the intensity ratio in the RCP focal spot with RCP illumination over the intensity with LCP illumination lies within 15 to 20 dB, and similarly for the polarization contrast of LCP, limited by the detection of our photodetector.

## Conclusion

We have presented the design and realization of a chiral meta-lens that can simultaneously and separately image both circular polarizations of a scene without suffering from the efficiency trade-off of geometric phase lenses. The design has a high polarization contrast of up to 20 dB without efficiency trade-off and thus provides the bases for a highly compact polarization imaging system for applications in remote-sensing, atmospheric science, medicine and biological imaging. The design can readily be adapted to other wavelength ranges so long as low-loss, high index subwavelength elements with tunable birefringence can be realized.

## Electronic supplementary material

Supplementary Information
