# Supplementary material for: High-efficiency chiral meta-lens
Source: Sci Rep. 2018 May 8;8:7240. doi: 10.1038/s41598-018-25675-3 (PMC5940891; doi:10.1038/s41598-018-25675-3)
Supplement: Supplementary file 1 — Supplementary Information [file 41598_2018_25675_MOESM1_ESM.docx]

**Supplementary Information for:**

**High-efficiency chiral meta-lens**

Benedikt Groever^1^, Noah A. Rubin^1^, J. P. Balthasar Mueller^1^, Robert C. Devlin^1^
and Federico Capasso^1^

^1^John A. Paulson School of Engineering and Applied Sciences, Harvard University, 29 Oxford Street,

^*^Corresponding author:[capasso@seas.harvard.edu](file:///C:\\Users\\bgroe\\Home\\Library\\Containers\\com.apple.mail\\Users\\ASUS\\Downloads\\capasso@seas.harvard.edu)

# Supplementary Note1: Derivation of the phase profile

$$\sin\theta f$$

$$f$$

$$f$$

$$x$$

chiral meta-lens

$$\cos\left( \theta\right)f$$

Fig. S1: Ray diagram for chiral imaging with incident collimated light. LCP light focuses to the left. RCP light focuses to the right.

$$\varphi_{RCP}=-\frac{2\pi}{\lambda_{0}}\left[ \sqrt{y^{2}+{(x + \sin\theta f)}^{2}+ \left( \cos\theta f \right)^{2}} -f \right]$$

$$\varphi_{RCP}=-\frac{2\pi}{\lambda_{0}} \left[ \sqrt{y^{2}+x^{2}+ 2x f\sin\theta+ \left( \sin\theta f \right)^{2}+\left( \cos\theta f \right)^{2}} - f \right]$$

$$\varphi_{RCP}=-\frac{2\pi}{\lambda_{0}} \left[ \sqrt{r^{2}+f^{2}+ 2x f\sin\theta}-f \right]$$

# Supplementary Note 2: Taylor series expansion of the phase profile

$$\varphi_{RCP}=-\frac{2\pi}{\lambda_{0}} \left[ \sqrt{r^{2}+f^{2}+ 2x f\sin\theta}-f \right]$$

Assume: $r^{2}\ll f^{2}+ 2x f\sin\theta$, and use: $\sqrt{f^{2}+ 2x f\sin\theta}\sqrt{1+z^{2}}=\sqrt{f^{2}+ 2x f\sin\theta} \left( 1+ \frac{z^{2}}{2}-\frac{z^{4}}{8}+ O(z^{5}) \right)$

with: $z^{2}=\frac{r^{2}}{f^{2}+ 2x f\sin\theta}$

$$\varphi_{RCP}=-\frac{2\pi}{\lambda_{0}} \left[ \sqrt{f^{2}+ 2x f\sin\theta}+ \frac{r^{2}}{2\sqrt{f^{2}+ 2x f\sin\theta}}-\frac{r^{4}}{8\left( f^{2}+ 2x f\sin\theta\right)^{3/2}}-f \right]$$

Assume: $2xf sin \theta\ll f^{2}$, and use: $f \sqrt{1+z}=f \left( 1+ \frac{z}{2}+ O(x^{2}) \right)$ with z $=\frac{2x f\sin\theta}{f^{2}}$

$$\varphi_{RCP}=-\frac{2\pi}{\lambda_{0}} \left[ f+ \frac{x f\sin\theta}{f}+ \frac{r^{2}}{2\sqrt{f^{2}+ 2x f\sin\theta}}-\frac{r^{4}}{8\left( f^{2}+ 2x f\sin\theta\right)^{3/2}}-f \right]$$

Use: $f \sqrt{1+z}=f \left( 1+ \frac{z}{2}+ O(z^{2}) \right)$ with z $=\frac{2x f\sin\theta}{f^{2}}$ again:

$$\varphi_{RCP}=-\frac{2\pi}{\lambda_{0}} \left[ x\sin\theta+ \frac{r^{2}}{2 \left( f+x\sin\theta\right)}-\frac{r^{4}}{8\left( f^{2}+ 2x f\sin\theta\right)^{3/2}} \right]$$

Use: $\left( \frac{r^{2}}{2f} \right)\frac{1}{1+z}=\left( \frac{r^{2}}{2f} \right)\left( 1-z+ O\left( z^{2} \right) \right)$ with: z $=\frac{x\sin\theta}{f}$

$$\varphi_{RCP}=-\frac{2\pi}{\lambda_{0}} \left[ x\sin\theta+ \left( \frac{r^{2}}{2f} \right)\left( 1-\frac{x\sin\theta}{f} \right)-\frac{r^{4}}{8\left( f^{2}+ 2x f\sin\theta\right)^{3/2}} \right]$$

leading to:

$$\varphi_{RCP}=-\frac{2\pi}{\lambda_{0}} \left[ x\sin\theta- \frac{r^{2}}{2f^{2}}x\sin\theta+ \frac{r^{2}}{2f}-\frac{r^{4}}{8\left( f^{2}+ 2x f\sin\theta\right)^{3/2}} \right]$$

Similar for LCP light:

$$\varphi_{LCP}=-\frac{2\pi}{\lambda_{0}} \left[ -x\sin\theta+ \frac{r^{2}}{2f^{2}} x\sin\theta+ \frac{r^{2}}{2f}-\frac{r^{4}}{8\left( f^{2}- 2x f\sin\theta\right)^{3/2}} \right]$$

Gradient terms
Focusing terms

# Supplementary Note 3: Zeroth order coupling loss


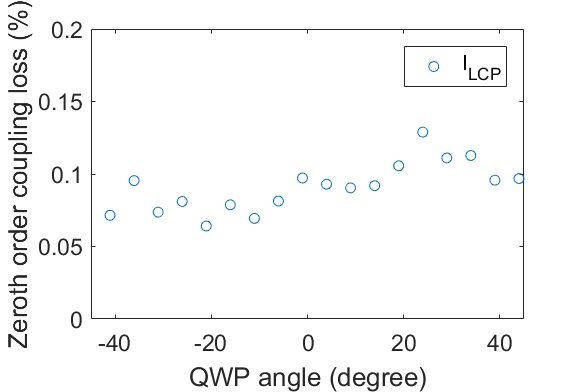


Fig. S2: Zeroth order coupling loss at different QWP angle

**Supplementary Note 4: Broadband behavior**

|  | **RCP incidence** | | **LCP incidence** | |
| --- | --- | --- | --- | --- |
|  | **RCP detector** | **LCP detector** | **RCP detector** | **LCP detector** |
| **500nm** | **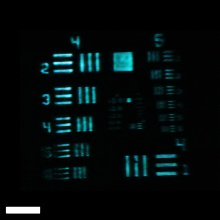** | **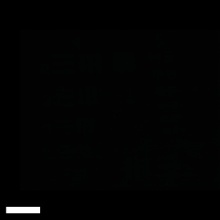** | **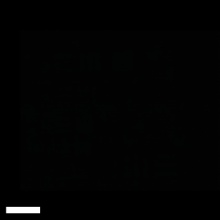** | **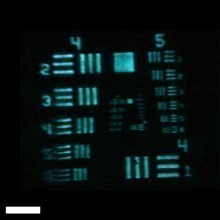** |
| **510nm** | **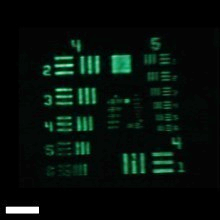** | **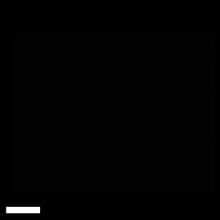** | **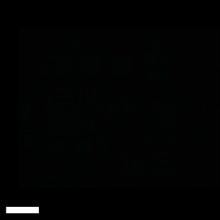** | **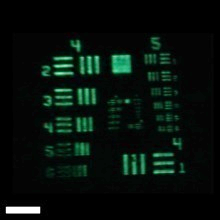** |
| **520nm** | **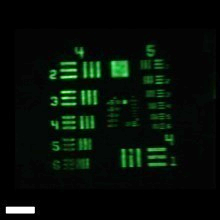** | **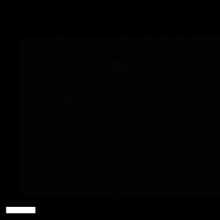** | **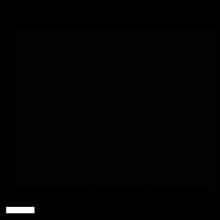** | **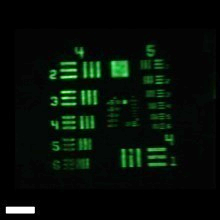** |
| **530nm** | **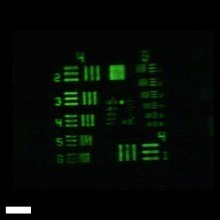** | **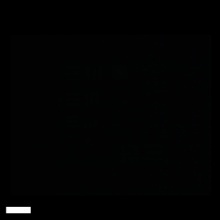** | **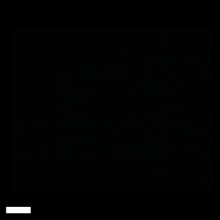** | **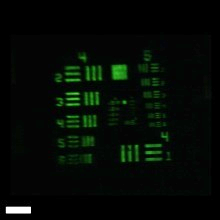** |
| **540nm** | **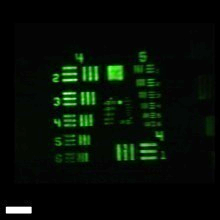** | **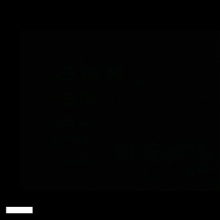** | **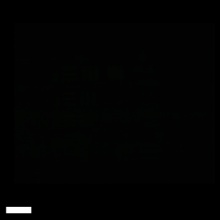** | **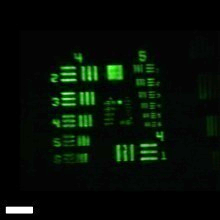** |
| **550nm** | **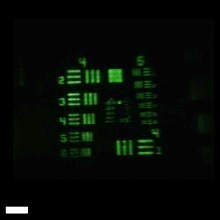** | **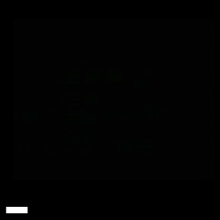** | **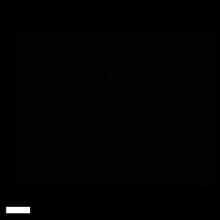** | **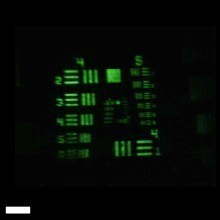** |
| **560nm** | **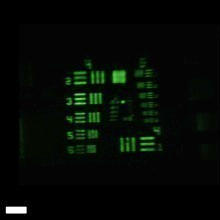** | **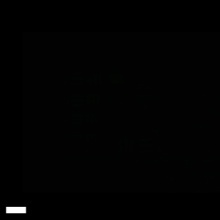** | **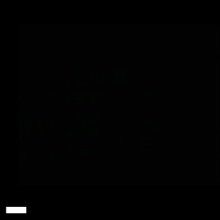** | **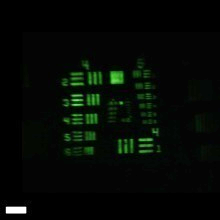** |
| **570nm** | **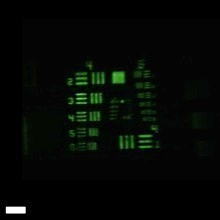** | **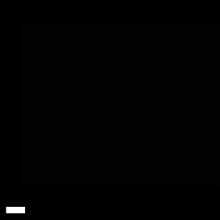** | **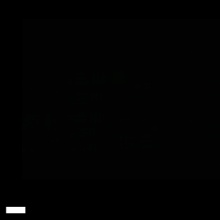** | **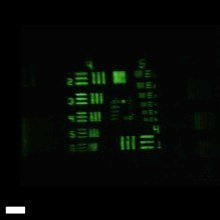** |
| **580nm** | **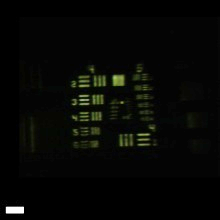** | **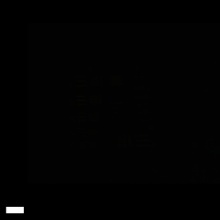** | **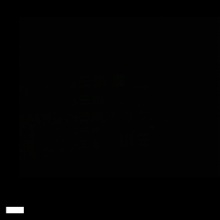** | **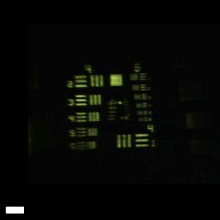** |
| **590nm** | **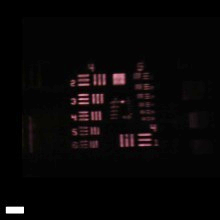** | **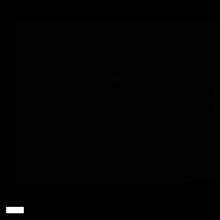** | **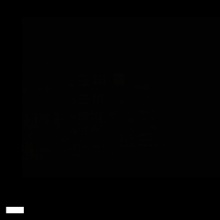** | **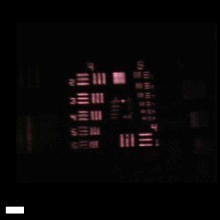** |
| **600nm** | **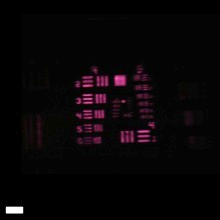** | **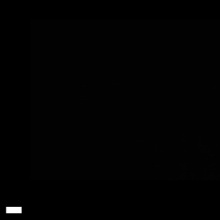** | **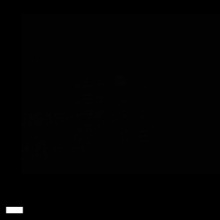** | **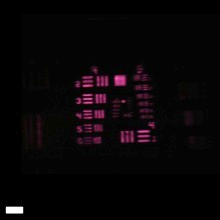** |
| **610nm** | **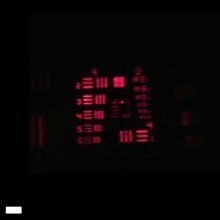** | **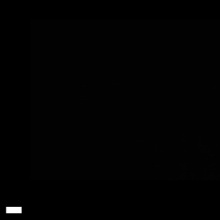** | **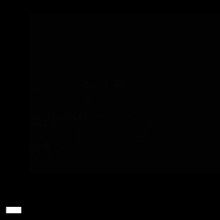** | **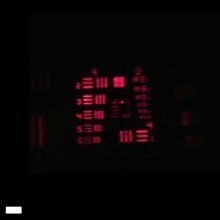** |
| **620nm** | **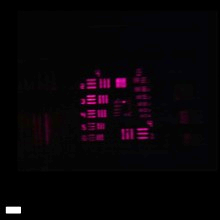** | **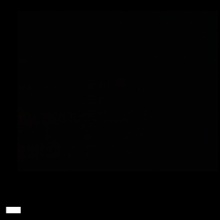** | **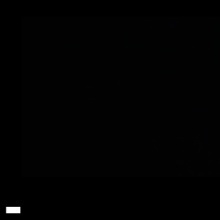** | **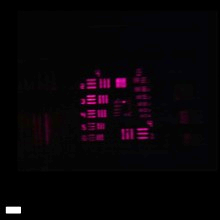** |

Fig. S3: Broadband imaging from 500 nm to 620 nm. Scale bar: 180 µm.

| **Wavelength (nm)** | **Image distance**  **(mm)** | **Focal length (mm)** | **Focal length uncertainty  (mm)** |
| --- | --- | --- | --- |
| 500 | 88.88 | 18.65 | 0.52 |
| 510 | 85.63 | 18.50 | 0.52 |
| 520 | 80.38 | 18.24 | 0.52 |
| 532 | 72.63 | 17.81 | 0.52 |
| 540 | 71.69 | 17.75 | 0.52 |
| 550 | 67.88 | 17.51 | 0.53 |
| 560 | 62.69 | 17.15 | 0.54 |
| 570 | 60.50 | 16.98 | 0.55 |
| 580 | 57.50 | 16.73 | 0.57 |
| 590 | 56.25 | 16.62 | 0.57 |
| 600 | 53.50 | 16.38 | 0.59 |
| 610 | 51.88 | 16.22 | 0.60 |
| 620 | 50.06 | 16.04 | 0.62 |

Table. S1: Imaging distance for each wavelength in Fig. S2. The object distance is fixed at 23.6 mm. The focal length and the corresponding error can be obtained through the lens equation.

The error in focal length can be calculated as following:

$$\sigma_{f}=\sqrt{\left( \frac{df}{di} \right)^{2}\sigma_{i}+\left( \frac{df}{do} \right)^{2}\sigma_{o}}$$

with

$$\frac{df}{di}=\frac{i \left( o+i \right)- o i}{\left( o+i \right)^{2}}$$

$$\frac{df}{do}=\frac{o \left( o+i \right)- o i}{\left( o+i \right)^{2}}$$

and

$\sigma_{i} =5$mm

$\sigma_{o}=0.75$mm

*i* is the image distance

*o* is the object distance

$\sigma_{i}$ is the error in the image distance
$\sigma_{o}$ is the error in the object distance


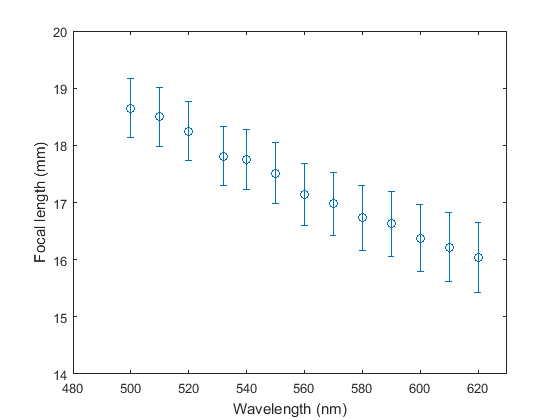


Fig. S4: Focal length at different wavelength.

The graph follows the expected behavior of a diffractive optical element: the focal length is inversely related to the wavelength. At the designed wavelength, the measured focal length is within the error of uncertainty of the designed focal length.

**Supplementary Note 5: RCP focusing efficiency**

**
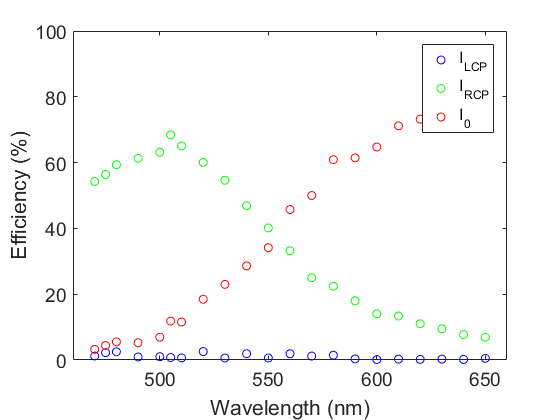
**

Fig. S5: RCP focusing efficiency – in analogy to the LCP focusing efficiency in Fig. 4(a).
